# Supplementary material for: Mapping the evolution of fertility support policies in China: A content and instrumental analysis
Source: PLoS One. 2025 Oct 9;20(10):e0332137. doi: 10.1371/journal.pone.0332137 (PMC12510515; doi:10.1371/journal.pone.0332137)
Supplement: S1 Appendix — (ZIP) [file pone.0332137.s001.zip › S1 Appendix. 226 original policy documents/25-国家卫生计生委关于开展新一轮全国计划生育优质服务先进单位创建活动的通知(FBM-CLI-4-269719).doc]

国家卫生计生委关于开展新一轮全国计划生育优质服务先进单位创建活动的通知

**发布部门：** [国家卫生和计划生育委员会(已撤销)](https://www.pkulaw.com/law/chl?Aggs.IssueDepartment=60319) [机构沿革](javascript:void(0))

**发文字号：**国卫指导发〔2016〕17号

**发布日期：**2016.05.03

**实施日期：**2016.05.01

**时效性：** [现行有效](https://www.pkulaw.com/law/chl?Aggs.TimelinessDic=01)

**效力级别：** [部门工作文件](https://www.pkulaw.com/law/chl?Aggs.EffectivenessDic=XE0303)

**法规类别：** [计划生育管理](https://www.pkulaw.com/law/chl?Aggs.Category=09705)

国家卫生计生委关于开展新一轮全国计划生育优质服务先进单位创建活动的通知
（国卫指导发〔2016〕17号）

各省、自治区、直辖市卫生计生委，新疆生产建设兵团人口计生委，解放军、武警部队计划生育领导小组办公室：
　　为深入贯彻《中共中央 [国务院关于实施全面两孩政策改革完善计划生育服务管理的决定](https://www.pkulaw.com/chl/8564756bae805261bdfb.html)》（中发〔2015〕40号，以下简称中央《决定》）和《[人口与计划生育法](https://www.pkulaw.com/chl/18c6c388fc14eaa9bdfb.html)》，全面提升计划生育工作水平，我委决定开展新一轮全国计划生育优质服务先进单位（以下简称新国优）创建活动。
　　**一、**充分认识开展新国优创建活动的重要意义
　　自2002年开展创建全国计划生育优质服务先进单位活动以来，各级党委政府和计划生育部门坚持以人为本、以技术服务为重点，加强计划生育服务体系建设，提升服务质量和管理水平，促进了工作思路和方法的转变，涌现出一大批先进典型，为落实计划生育基本国策、保障群众计划生育权益、推动计划生育事业创新发展发挥了重要作用。
　　党的十八届五中全会决定全面实施一对夫妇可生育两个孩子的政策，开展新国优创建活动，是实施好全面两孩政策、促进人口长期均衡发展的有力保障，是改革完善服务管理、提高计划生育工作能力和工作水平的有效载体，是增进家庭幸福、促进社会和谐、不断增强人民群众获得感和满意度的重要举措。

　　**二、**工作目标和基本原则
　　（一）工作目标。深入学习贯彻十八届五中全会和中央《决定》精神，坚持计划生育基本国策，以实施好全面两孩政策为重点，以改革完善计划生育服务管理为动力，健全管理体制，完善服务机制，强化基层基础，创建一批求真务实、成效明显、群众满意的新国优，以点带面，示范引领，推动实现计划生育治理体系和治理能力现代化。
　　（二）基本原则。
　　一是以人为本，强化服务。尊重群众主体地位，依法行政，文明执法，维护群众计划生育合法权益。以群众需求为导向，不断提升计划生育优质服务水平。
　　二是严格标准，分级负责。坚持创建标准的引导作用，严格评选，好中选优。建立县级主创、市级推荐、省级评审、国家命名的工作机制，落实领导和监督责任。
　　三是分类指导，协同发展。将国家要求与本地实际紧密结合，着力解决薄弱环节和难点问题。加强与相关部门的政策衔接，形成推进创建活动的合力。
　　四是总量控制，动态管理。3年为一评选周期，每一周期各省（区、市）评选出的新国优比例不超过应参评单位总数的20%。连续两个周期被评为新国优的，荣誉称号可顺延一个周期。

　　**三、**主要内容
　　（一）落实党政责任。要把思想和行动统一到中央决策部署上来，把实施全面两孩政策、改革完善计划生育服务管理作为重要工作纳入经济社会发展总体规划。党政主要领导亲自抓、负总责，坚持和完善计划生育工作领导小组制度，落实目标管理责任制和一票否决制，确保责任到位、措施到位、投入到位。
　　（二）加强部门协作。计划生育工作领导小组成员单位要履职尽责，进一步健全齐抓共管计划生育的工作格局。完善实施全面两孩政策的配套措施，重点解决好公共服务保障、执法协调、信息互通等问题。增强家庭发展能力，建立完善生育支持、幼儿养育、青少年发展等家庭发展政策，鼓励按政策生育。
　　（三）做好宣传倡导。深入宣传人口国情和基本国策，宣传实施全面两孩政策、促进人口长期均衡发展的重大意义。加强政策解读，正确引导舆论。深入开展婚育新风进万家活动和关爱女孩行动，普及优生优育、生殖健康知识，倡导科学、文明、进步的婚育观念。
　　（四）依法规范管理。实行生育登记服务制度，优化办事流程，全面推行网上办事。依法行政，文明执法，规范社会抚养费征收管理，维护良好的生育秩序。加强计划生育政务公开，深入推进诚信计生和群众自治。完善宣传倡导、依法管理、优质服务、政策推动、综合治理的计划生育长效工作机制。
　　（五）提升服务水平。推进优生优育全程服务，落实免费孕前优生健康检查，加强孕产期保健服务和出生缺陷综合防治。推进妇幼保健计划生育服务机构标准化建设和规范化管理，加强孕产妇与新生儿危急重症救治能力建设。全面实行避孕方法自主知情选择。大力推进流动人口基本公共卫生计生服务均等化。
　　（六）完善扶助保障。加大对计划生育家庭扶助力度，落实各项奖励和扶助政策。相关民生政策对政策调整前的独生子女家庭和农村计划生育双女家庭予以倾斜。关怀计划生育特殊家庭，妥善解决他们的生活照料、养老保障、大病治疗和精神慰藉等问题。
　　（七）强化信息支撑。健全县乡村三级出生监测网络，实现生育登记、孕产期保健、住院分娩、出生医学证明、儿童预防接种等信息共享。推动户籍管理、婚姻、人口健康、教育、社会保障等信息互联互通。运用信息化手段，优化工作流程，提高服务效率。
　　（八）巩固基层网络。优化整合卫生计生资源，稳定和加强县、乡级计划生育工作力量，加强队伍和能力建设。妥善解决好村级计划生育专干的报酬待遇、养老保障等问题。建立健全卫生和计划生育综合监督行政执法体系。加强计划生育协会组织建设，充分发挥生力军作用。

　　**四、**扎实推进新国优创建活动
　　（一）加强组织领导。各省（区、市）卫生计生委要把创建活动作为贯彻落实中央《决定》的重要抓手，主要领导亲自抓，分管领导具体抓，明确相关处室责任，统筹协调，强力推进。以县（市、区）为重点，上下联动，广泛深入地开展创建活动。
　　（二）制定创建方案。各省（区、市）卫生计生委要根据本通知精神，结合本地实际，制定具体创建活动方案，明确目标任务，细化评估内容，严格评审程序，建立激励机制，强化保障措施，推动工作落实。
　　（三）抓好组织实施。加强创建活动全过程的调研指导，总结和宣传先进典型。组织好评选活动，坚持好中选优，把求真务实、成效明显、群众满意的县（市、区）评选出来，确保创建活动的质量和生命力。
　　（四）强化督导检查。严明工作纪律，避免突击性创建，防止创建活动走过场。对弄虚作假、违规评审等行为，一经查实，严肃处理。各省（区、市）卫生计生委要加强督导，我委将不定期组织检查。
　　各省（区、市）卫生计生委要高度重视、认真组织，评选出自2014年以来在卫生计生机构改革、调整完善生育政策、全面推进依法行政、深化服务管理改革等方面取得显著成效的先进县（市、区）。2016年6月15日前将创建活动方案报送我委，10月31日前将拟命名的新国优名单及创建活动报告报送我委，年底前我委将命名第一批新国优。
　　附件：1. 全国计划生育优质服务先进单位创建活动管理办法（试行）
　　2.全国计划生育优质服务先进单位创建活动评估重点（2016年版）

国家卫生计生委
2016年5月3日

　　附件1

全国计划生育优质服务先进单位创建活动管理办法
（试行）

　　**第一条**　根据《中共中央 [国务院关于实施全面两孩政策改革完善计划生育服务管理的决定](https://www.pkulaw.com/chl/8564756bae805261bdfb.html)》（中发〔2015〕40号），为规范新一轮全国计划生育优质服务先进单位（以下简称新国优）创建活动，制定本办法。

　　**第二条**　新国优是国家卫生计生委授予的在计划生育工作中求真务实、成效明显、群众满意，能够发挥示范引领作用的县（市、区）的荣誉称号。

　　**第三条**　新国优创建活动以县（市、区）为单位开展，3年为一评选周期，期满后荣誉称号自动终止。连续两个周期被评为新国优的，荣誉称号可顺延一个周期。

　　**第四条**　新国优名额实行总量控制，每一周期各省（区、市）评选出的新国优比例不超过应参评单位总数的20%。

　　**第五条**　国家卫生计生委统筹协调和指导创建活动，制订管理办法和评估重点，组织公示和命名表彰。
　　省级卫生计生委组织本辖区创建活动，制订创建活动方案，细化评估内容，负责评审和监督。

　　**第六条**　新国优的评选按照县级申报、市级推荐、省级评审、国家命名的程序进行。
　　（一）县级申报。具备申报资格的县（市、区），以县级人民政府名义向设区的市级卫生计生行政部门提交申报报告。
　　（二）市级推荐。设区的市级卫生计生行政部门对提出申请的县（市、区）进行审核，本着好中选优的原则，向省级卫生计生委推荐。
　　（三）省级评审。省级卫生计生委对申报县（市、区）进行评审，确定新国优名单，公示后上报国家卫生计生委。
　　（四）国家命名。国家卫生计生委通过政府网站等渠道，对候选名单公示10个工作日，接受社会评议和监督。对有反映问题的，及时组织核查。通过公示和核查的，授予荣誉称号，发文通报，颁发奖牌。

　　**第七条**　省级卫生计生委结合本地实际，建立健全相应的激励机制，总结推广新国优的先进经验，把创建活动不断引向深入。

　　**第八条**　有下列情形之一的，由省级卫生计生委核实，撤销其荣誉称号，并报国家卫生计生委备案。
　　（一）发生重大违法行政案件的；
　　（二）发生严重技术服务事故的；
　　（三）工作水平严重滑坡的；
　　（四）统计数据严重失实的。
　　被撤销荣誉称号的，不得参加下一周期新国优的申报和评选。

　　**第九条**　省级卫生计生委每年向国家卫生计生委报送创建活动报告。对在创建活动中组织指导不力、搞形式、走过场的，国家卫生计生委将予以通报，并酌情核减下一周期新国优评选名额。

　　**第十条**　本办法由国家卫生计生委负责解释。

　　**第十一条**　本办法自2016年5月1日起施行。
　　附件2
　　全国计划生育优质服务先进单位创建活动评估重点
　　（2016年版）

| 一、党政领导 | 深入学习中央《决定》，提出贯彻落实的具体措施。党政主要领导亲自抓、负总责，定期召开计划生育工作领导小组会议，落实目标管理责任制和一票否决制。计划生育工作经费本级投入足额到位，管理规范。 |
| --- | --- |
| 二、部门协作 | 各相关部门认真履职，实施全面两孩政策的配套措施完善，公共服务保障、执法协调、信息互通等方面有机衔接。鼓励按政策生育的家庭发展支持体系基本建立，计划生育多元共治格局基本形成。 |
| 三、宣传倡导 | 宣传倡导活动经常化，深入开展第五阶段婚育新风进万家活动和关爱女孩行动，群众计划生育政策和优生优育知识知晓率较高。创建活动形成良好氛围，群众满意度较高。 |
| 四、依法管理 | 实行生育登记服务制度，优化办事流程，全面推行网上办事。计划生育政务信息及时公开。对群众诉求及时协调处理，无信访积案，无重大违法行政行为。出生人口性别比基本正常或出现稳步下降的态势。深入推进诚信计生和村（居）民自治。 |
| 五、生育服务 | 落实优生优育全程服务，妇幼保健计划生育服务机构建设标准化、管理规范化。免费孕前优生健康检查项目目标人群覆盖率达85%。全面落实自主知情选择，避孕药具保障供应。流动人口基本公共卫生计生服务目标人群覆盖率达85%。 |
| 六、扶助保障 | 各项奖励和扶助政策全部落实，相关民生政策对政策调整前的独生子女家庭和农村计划生育双女家庭予以倾斜。对计划生育特殊家庭的生活照料、养老保障、大病治疗和精神慰藉等工作落实到位、成效明显。 |
| 七、信息支撑 | 卫生计生以及跨部门信息安全共享机制基本建立，生育登记、出生人口、奖励扶助等信息及时采集、动态更新，准确率达95%。流动人口婚育信息异地查询、反馈率达90%。 |
| 八、基层网络 | 基层计划生育行政管理、技术服务、群众工作网络健全，基础工作扎实。卫生计生资源优化整合，监督执法体系完善。妥善解决好村级计划生育专干的报酬待遇、养老保障等问题。 |

© 北大法宝：（[www.pkulaw.com](https://www.pkulaw.com/)) 专业提供法律信息、法学知识和法律软件领域各类解决方案。北大法宝为您提供丰富的参考资料，正式引用法规条文时请与标准文本核对。
欢迎查看所有[产品和服务](https://www.pkulaw.com/helps/2.html)。法宝快讯：[如何快速找到您需要的检索结果？](https://www.pkulaw.com/helps/70.html)[法宝](https://www.pkulaw.com/helps/69.html)V6有何新特色？

扫描二维码阅读原文

**原文链接：**<https://www.pkulaw.com/chl/29494c738d9d3316bdfb.html>
